# Ekbom Syndrome: A Case Report

**DOI:** 10.1192/bjo.2022.350

**Published:** 2022-06-20

**Authors:** Nadine Absy, Nur Saleh, Dima Al Sharman

**Affiliations:** Jordan University of Science and Technology, Irbid, Jordan

## Abstract

**Aims:**

Ekbom's syndrome (ES), also called delusional parasitosis, is a condition where the patient has an unshakable belief and a perception of being infested with parasites. ES is thought to mainly affect postmenopausal females and because patients are usually mono-symptomatic, they usually seek care from dermatologists. It is advocated to form a liaison between dermatology and psychiatry to ensure a full range of differential diagnoses, in order to form the most suitable management plan.

**Methods:**

Case report

**Results:**

An 87-year-old widow was referred to the outpatient psychiatric clinic of King Abdullah University Hospital by a dermatologist because of generalized chronic pruritus that she believes is caused by a bug infestation. The symptoms started one year prior to presentation (soon after an ischemic stroke) with the perception that macroscopic parasites were crawling over her body, biting her face, head, and hands, and entering her eyes. She tried various strategies to eradicate the parasites with no benefit. Psychiatric examination findings included hypochondriac delusional ideas and dysphoria. When her general medical condition and her medications were reviewed, it was found that she had been diagnosed with hypertension and ischemic heart disease. She was taking anti-hypertensive drugs and blood thinners. After haloperidol 5 mg daily was added, she had a progressive clinical improvement.

**Conclusion:**

ES is a neuropsychiatric syndrome that can follow primary psychotic or depressive disorders, dementia, or other organic diseases. Consultation-liaison by psychiatrists and dermatologists will be useful to assure timely referral. Better awareness of such an illness by general physicians, early recognition, good rapport, and empathic treatment are the cornerstones of management in such cases.

